# Public knowledge and beliefs about dementia risk reduction: a national survey of Australians

**DOI:** 10.1186/1471-2458-14-661

**Published:** 2014-06-28

**Authors:** Ben J Smith, Suha Ali, Henry Quach

**Affiliations:** 1 School of Public Health and Preventive Medicine, Monash University, Lev 3, 89 Commercial Rd, Melbourne 3004, Australia; 2 Alzheimer’s Australia (Victoria), 155 Oak Street, Parkville 3052, Australia

**Keywords:** Dementia, Cognitive health, Health behaviours, Health promotion

## Abstract

**Background:**

With the dramatically increasing contribution of Alzheimer’s Disease and other forms of dementia to the global burden of disease, countries are being urged to address this as a public health priority. This study investigated whether Australian adults recognise this as an important health issue, and hold beliefs and knowledge that are consistent with recommendations concerning dementia risk reduction. This research was undertaken to guide national brain health awareness and education strategies.

**Methods:**

A cross-sectional telephone survey was undertaken of 1,003 Australians aged 20–75 years. This measured the importance placed on dementia, beliefs and confidence related to risk reduction, knowledge of risk reduction methods, and the perceived age-relevance of these. In analysis the data were stratified by sex, age, educational attainment, household income, language preference and previous exposure to dementia. Multivariable logistic regression was undertaken to identify variables independently associated with beliefs and knowledge.

**Results:**

People aged 60 years and over identified dementia as very important (17.2%) more often than those aged 40–59 years (5.1%) or 20–39 years (2.1%). While 41.5% of respondents believed the risk of dementia could be reduced, 26.9% were very confident that they could achieve this. Mental activity (57.1%) was identified as beneficial much more often than physical activity (31.3%), healthy eating (23.3%) and other cardiovascular health behaviours. Women, people of English-speaking origin, and those having contact with a person with dementia, showed better knowledge of several health behaviours.

**Conclusions:**

Growing attention is being given to population risk reduction to combat the dramatic increase in the burden of disease due to dementia. In Australia many people do not yet hold beliefs and knowledge that support this, which highlights the need for concerted awareness raising that dementia is not an inevitable aspect of ageing, and for education about the role of vascular health in dementia risk reduction.

## Background

Alzheimer’s disease and other forms of dementia are among the most rapidly increasing threats to public health globally. In 2012 the Global Burden of Disease Project reported that over the two decades from 1990 until 2010 the total disability adjusted life years due to Alzheimer’s disease and other forms of dementia had risen by 99.3% [1], an increase of 53.3% per 100,000 persons. Over this period the age standardised death rate due to these conditions was estimated to have risen by 95.4% [2]. Such dramatic rises have been driven by population aging and the epidemiological transition that is underway in many low and middle income countries [3].

Countries in Western Europe, North America and the developed region of the Western Pacific have been identified as having the highest prevalence of dementia [4]. In Australia, dementia was ranked as the third leading cause of death in 2010 and the fourth highest contributor to the total burden of disease in 2011 [5]. It is projected that the number Australians with some form of dementia will triple by the year 2050 [5]. Similar projections have been made for the United States [6], the United Kingdom [7] and nations across Europe [8].

As a result of the marked increase in the burden of disease due to dementia the World Health Organisation has called for nations to place this issue on their public health agendas. Raising awareness and reducing stigma, improving dementia care and services, and providing support for caregivers are important areas of action [9]. There is also growing support for a proactive approach, to reduce the incidence and delay the onset of dementia through health promotion efforts directed at whole populations [10-12].

Current evidence concerning the aetiology of Alzheimer’s disease and dementia indicates that, apart from age and genetic characteristics, there are potentially modifiable biomedical, behavioural, psychological and social factors associated with disease risk. Biomedical factors that are consistently recognised are diabetes, hypertension, hyperlipidaemia and obesity [13,14]. Smoking, physical activity, dietary intake and levels of alcohol consumption are behaviours that are related to dementia risk [15,16]. Psychological and social factors that have been reported to be associated with age-related cognitive decline are depression, cognitive activity and social contact [17,18]. Recent epidemiological studies which have reported a decline in the prevalence of dementia among the elderly, compared with earlier cohorts, suggest that reductions in risk factors at the population level and improvements in standards of living and health care can lower the burden of disease due to this condition [19,20].

Determining the relative contribution of the risk factors to different forms of dementia is a research priority and the role of some factors (e.g., alcohol intake), and their interaction with genetic characteristics, is still being explored [21]. Notwithstanding this, the global increases in the Alzheimer’s Disease and dementia, and the observed associations with modifiable factors has prompted researchers and advocates to recommend that population level risk reduction strategies be instigated by government and non-government agencies [22]. A growing number of organisations are heeding this call, demonstrated by initiatives such as the *Healthy Brain Initiative* in the US [23], the *Your Brain Matters* campaign in Australia [24], the *National Memory Programme* in Finland [25] and the *Forget Me No*t education program in Ireland [26]. This arena of activity has brought with it a need for new evidence, about the prevailing knowledge, beliefs and behaviours concerning dementia and the potential to maintain cognitive health among different population segments [27].

Studies of public knowledge and beliefs towards Alzheimer’s disease and other forms of dementia have focused predominantly upon perceived severity and personal susceptibility to these diseases, and understanding of symptoms and treatment [28-32]. A review of research about public perceptions of cognitive health in the US found that 3 of 10 studies examined beliefs about lifestyle and brain health, or the potential to reduce the risk of dementia [33]. Of these, the Brain Health Poll undertaken by the American Society on Ageing and Metlife Foundation [34] was the most extensive, investigating beliefs about whether brain health could be improved and the actions that were believed to beneficial for this purpose. A more recent national survey in Australia [35] investigated similar questions, with a focus upon dementia risk reduction rather than brain health improvement. Other studies have been undertaken with selected population groups (e.g., older people, carers), used convenience samples, and measured knowledge of a narrower range of risk factors [36-39].

With leading authorities advocating for a public health approach towards dementia there is a need to monitor the extent to which populations have also adopted this perspective. Up-to-date data is required about public knowledge and beliefs, including the importance placed on dementia, the potential for risk reduction, the perceived age-groups for whom this is relevant, the behaviours that should be undertaken, and confidence that these will produce beneficial outcomes. This purpose of the study reported here was to investigate these factors among a cross-section of Australian adults, to determine the prevalence of risk reduction knowledge and beliefs and differences between socio-demographic groups. The longer term objective of this research was to inform the design and targeting of national brain health initiatives.

## Methods

### Study population and sampling

An Australia-wide sample of persons aged 20–75 years was recruited. Participants were required to speak English and not be affected by a mental impairment that reduced their ability to answer the survey questions.

Sampling was undertaken using random digit dialling, by a social research organisation with extensive experience in epidemiological and population studies. Because of the growing number of persons who use a cell phone in preference to a land line, a dual sampling frame was developed that comprised 70% landline numbers and 30% cell phone numbers. In order to improve representativeness, when households were contacted a request was made to speak with the individual who had the next birthday.

Ethical approval for the conduct of the study was given by the Monash University Human Research Ethics Committee (approval no. CF12/2427-2012001309).

### Procedures

Surveys were conducted by trained interviewers over three weeks, from August to September 2012. In order to determine eligibility and complete recruitment, up to six calls were made to establish contact and a further four to achieve completed interviews. The length of surveys was approximately 10 minutes.

### Measures

To investigate knowledge and beliefs related to dementia, straightforward questions with good face and content validity were used, several of which have been used in previous population studies. In order to measure the personal importance that respondents placed upon dementia as a health issue, the questions used in the US Brain Health Poll [34] were adopted. These were two open-ended questions which asked respondents to nominate the first and second health issues that it is most important for them to have information about.

Several questions, which had been used in previous surveys commissioned by Alzheimer’s Australia [40], were asked to determine whether respondents believed that the risk of developing dementia could be reduced, and their knowledge of the behaviours that are beneficial for risk reduction. The first of these measures required individuals to respond, using a 5-point Likert scale, to the question: “Thinking now about dementia, which has many causes including Alzheimer’s disease, please tell me if you personally agree or disagree that it is possible to reduce the risk of a person developing dementia”. Following this an open-ended question was asked: “As far as you are aware, what do you think a person can do to help reduce the risk of developing Alzheimer’s disease or another form of dementia?”. Up to five responses were elicited by means of prompting.

To determine when respondents believed it is beneficial to commence action to reduce dementia, they were asked: “At what age do you think people should start to take action to reduce their risk of Alzheimer’s disease or another form of dementia?” A list of age ranges (<18 years, 18–29 years etc.) were provided to choose from. In order to measure their personal confidence that they can take action to reduce their own risk of dementia respondents were asked: “How would you rate your confidence that you can take action now that will reduce your risk of Alzheimer’s disease or another form of dementia?” A four-point rating scale was given, ranging from very confident, to not confident at all.

The demographic information collected included gender, age, education level, household income and language spoken at home. Respondents were also asked if they had ever had personal contact with a person with dementia.

### Statistical analysis

The sample was weighted to the age and gender distribution of the Australian population. In bivariate analysis, the Chi-squared statistic was used to compare dementia beliefs and knowledge between different strata of respondents. The independent variables in these analyses were: gender; age group (20–39 years, 40–59 years, 60–75 years); educational attainment (up to year 10, high school or technical college, University); yearly household income (less than $40,000, $40,000-$99,000, $100,000 and over); language spoken at home (English, other), and; prior contact with a person with dementia (yes, no). Backwards step-wise logistic regression modelling was undertaken, including those variables where bivariate relationships were observed, to identify those independently associated with dementia beliefs and knowledge. A maximum P-value of 0.1 was adopted to determine retention of variables at each step of modelling. Analyses were carried out using SPSS V19.0.

## Results

### Respondent characteristics

In total 1,003 interviews were carried out with a cooperation rate, among eligible persons contacted, of 58%. The characteristics of the respondents are shown in Table 1. The mean age was 47.6 years (SD 18.44) and most were aged in the 40–59 year age group (39.1%). A higher proportion of respondents were women (57.1%). Just over 40% were educated at the university level and the majority were in full-time or part-time work (64.6%). Income groups were evenly spread between those earning $100,000 or more (30.0%), those earning less than $40,000 (29.6%), and those in the middle income range (40.4%). Just under 15% of respondents spoke a language other than English at home, and almost three-quarters reported prior contact with a person suffering from Alzheimer’s disease or other forms of dementia.

**Table 1:** Characteristics of survey respondents

**Characteristics**	**Unweighted**	**Weighted**
Mean age (SD)	47.6 (18.44)	46.2 (18.27)
Age groups		
20-39 yrs	299 (30.1%)	382 (38.3%)
40-59 yrs	397 (39.9%)	352 (35.3%)
60+ year	298 (30.0%)	263 (26.4%)
Sex		
Men	429 (42.9%)	491 (49.1%)
Women	571 (57.1%)	509 (50.9%)
Education level*		
Completed up to year 10	214 (21.6%)	202 (20.6%)
Completed year 12/TAFE	373 (37.7%)	369 (37.5%)
Completed University	402 (40.2%)	413 (42%)
Work status		
Full time/Part time work	643 (64.6%)	645 (64.9%)
Not working	352 (35.4%)	349 (35.1%)
Income groups		
Up to $39,999	245 (29.6%)	265 (31.8%)
$40,000 to $99,999	334 (40.4%)	319 (38.4%)
$100,000 or more	248 (30.0%)	248 (29.8%)
Language		
English	850 (85.3%)	839 (84.3%)
Other	146 (14.7%)	156 (15.7%)
Contact with dementia		
Yes	730 (73%)	713 (71.3%)
No	270 (27%)	287 (28.7%)

*14 people did not respond, so total percent is less than 100.

Table 1 shows the unweighted characteristics of respondents in the completed survey, and the profile of the sample after weighting to the sex and age distribution of the Australian population.

### Prevalence of knowledge and beliefs about dementia risk reduction

As shown in Table 2, 7.2% of survey respondents rated dementia as one of the two most important health issues of concern for them, which made it the sixth ranked condition after heart disease, stress, obesity, diabetes and prostate cancer. Overall, a higher proportion of women than men (9.0% vs 5.3%) rated dementia as of high importance to them. Table 2 shows that respondents aged 60 years and over ranked dementia as the third most important issue (17.2% nominated it), whereas among those aged 40–59 years it was ranked eighth (5.2%), and for those aged 20–39 years it ranked fifteenth (2.2%).

**Table 2:** Most important health issues identified by respondents, by age group, with ranking of dementia relative to others

**Rank**	**Total**	**%**	**18-39 years**	**%**	**40-59 years**	**%**	**60+ years**	**%**
1	Heart disease	22.5	Obesity	19.3	Heart disease	28.9	Heart disease	32.0
2	Stress	22.5	Heart disease	10.4	Stress	28.9	Stress	32.0
3	Obesity	11.5	Stress	10.4	Diabetes	11.2	Dementia	17.2
4	Diabetes	9.3	Alcohol	8.1	Prostate cancer	11.2	Arthritis	13.6
5	Prostate cancer	9.0	Skin cancer	8.0	Breast cancer	10.5	Diabetes	10.5
	Dementia (6)*	7.2	Dementia (15)*	2.2	Dementia (8)*	5.2		

*Ranking of dementia relative to other issues.

Table 3 shows that 41.5% of respondents strongly believed that the risk of dementia could be reduced, while just over one-quarter (26.9%) were very confident that they could take action to lower their personal risk of dementia. A lower proportion of women than men strongly agreed that the risk can be reduced, while a higher proportion of those with prior contact with a person with dementia held this belief compared with those who reported no prior contact. Most respondents believed that action to reduce dementia risk should commence before the age of 40 years; but there was a lower proportion of persons aged 60 years and over who held this belief compared to the younger age groups. Persons with a university level education and those in the lowest income bracket most frequently reported that efforts to lower dementia risk should begin before the age of 40 years.

**Table 3:** Beliefs about the reduction of dementia risk

	**Dementia risk reduction**		**Age groups for action**		
	**Strongly agree**	**Very confident**	**<40 yrs**	**40-59 yrs**	**60+ yrs**
Total	41.5	26.9	57.1	37.6	5.4
Sex					
Men	46.0**	30.7**	57.2	37.8	5.0
Women	37.0**	23.3**	56.9	37.3	5.8
Age group					
20-39 yrs	42.0	27.2	65.7***	32.7***	1.6***
40-59 yrs	39.6	23.5	56.3***	39.0***	4.6***
60+ yrs	42.9	30.8	44.4***	43.2***	12.4***
Education					
≤ year 10	43.8	26.7	52.2*	40.9*	7.0*
High School/TAFE	40.9	26.2	56.7*	35.7*	7.6*
University	41.1	26.8	58.8*	38.4*	2.8*
Income					
<$40 k	42.9	28.3	58.5*	34.4*	7.1*
$40-99 k	38.8	23.5	55.1*	42.2*	2.6*
$100+	43.9	23.4	53.4*	43.1*	3.4*
Language					
English	41.5	26.2	56.9	37.8	5.3
Other	41.0	30.8	57.0	37.6	5.4
Contact with dementia					
Yes	44.1*	27.0	57.2	37.9	4.9
No	34.6*	26.6	56.7	36.7	6.5

Chi-squared - *p < 0.05; **p < 0.01; ***p < 0.001.

The action that respondents most often identified as valuable for lowering dementia risk was mental activity, which was followed in levels of recognition by physical activity, a healthy diet, and social activity (Table 4). Moderate alcohol consumption and non-smoking were least frequently identified as beneficial for dementia prevention.

**Table 4:** Knowledge of behaviours that are beneficial for dementia risk reduction

	**Mental activity**	**Social activity**	**Physical activity**	**Healthy diet**	**Moderate alcohol**	**Non-smoking**
Total	57.1	12.1	31.3	23.3	5.1	3.1
Sex						
Men	51.5**	8.9**	29.9	18.9**	6.3	3.5
Women	62.4**	15.3**	32.6	27.5**	3.9	2.8
Age group						
20-39 yrs	51.3*	8.1**	27.7	22.8	6.6**	4.5
40-59 yrs	59.9*	12.2**	31.5	26.7	6.5**	2.8
60+ yrs	61.5*	17.9**	36.5	19.8	0.8**	1.1
Education						
≤ year 10	57.4	11.3**	34.7	18.8	3.0	1.5
High School/TAFE	57.5	8.4**	28.7	24.1	4.7	3.0
University	58.1	16.2**	32.7	25.7	6.1	4.1
Income						
<$40 k	53.6	11.3	35.1	23.0	1.1*	1.1
$40-99 k	58.8	12.5	31.7	23.4	5.9*	3.8
$100+	63.7	10.9	28.2	27.4	6.9*	3.6
Language						
English	60.7***	12.2	32.7*	25.3**	5.5	3.0
Other	37.8***	12.8	23.7*	14.1**	3.2	3.8
Contact with dementia						
Yes	61.9***	13.4*	33.9**	26.3***	4.8	3.4
No	45.1***	8.7*	24.7**	16.0***	5.9	2.4

Chi-squared - *p < 0.05; **p < 0.01; ***p < 0.001.

Women more frequently identified mental activity, social activity and a healthy diet as beneficial for dementia risk reduction than men. Respondents aged 60 years and over most often reported that mental activity and social activity are beneficial, whereas the highest proportion who recognised moderate alcohol consumption as beneficial were aged 20–39 years. Those with a university level education most often reported that social activity is beneficial for dementia risk reduction, and those in the highest income bracket most often recognised the value of moderate alcohol consumption. A higher proportion of respondents who spoke English at home recognised the value of mental activity, physical activity and a healthy diet. Respondents with prior contact with a person suffering from dementia also more frequently reported that these three behaviours are beneficial, and that social activity can assist in risk reduction.

### Multivariable analysis of factors associated with dementia beliefs and knowledge

Table 5 shows that although women were more likely than men to consider dementia to be a personal health priority (adjusted odds ratio (OR) 1.88, 95% confidence interval (CI), 1.07-3.31), they were less likely to believe that the risk of dementia could be reduced or to express confidence that they could achieve this outcome for themselves. Respondents aged 60 years and over were significantly more likely to consider dementia to be a very important health issue (OR 14.17, 95% CI 5.67-35.39) than those in the youngest age group, but were less likely to believe that risk reduction should start before the age of 40 years. Table 5 also shows that those from households in the top income category had the lowest adjusted odds of believing that risk reduction action should start in early adulthood, and those who have had contact with a person suffering from dementia had the highest odds of believing that the risk of dementia can be reduced.

**Table 5:** Demographic and social factors independently associated with risk reduction beliefs

	**Dementia rated as important**		**Believe risk can be reduced**		**Very confident can lower risk**		**Believe action should start at <40 yrs**	
	**Adj OR**	**95% CI**	**Adj OR**	**95% CI**	**Adj OR**	**95% CI**	**Adj OR**	**95% CI**
Sex								
Men (ref)								
Women	1.88	1.07-3.31	0.69	0.53-0.90	0.69	0.52-0.91		
Age								
20-39 (ref)									
40-59	3.29	1.23-8.76					0.66	0.48-0.92	
60+	14.17	5.67-35.39					0.32	0.21-0.48	
Income									
<$40 k (ref)									
$40-99 k								0.69	0.48-1.00
$100 k+								0.58	0.39-0.86
Contact w/-dementia									
No (ref)									
Yes			1.51	1.11-2.05					

Women were more likely than men to report knowledge of the value of mental activity, social activity and a healthy diet for dementia risk reduction (Table 6). Those aged 60 years had a significantly higher adjusted odds of recognising the benefits of social activity, but the lowest odds of identifying non-smoking (OR 0.28, 95% CI 0.09-0.91) as a beneficial action. Persons educated at the university level were more likely to recognise the benefits of being socially active for dementia risk reduction, compared to those educated up to the Year 10 level, and people in the middle and highest categories of household income were most likely to identify moderate alcohol consumption as beneficial. English speaking respondents had higher adjusted odds of identifying mental activity and a healthy diet as beneficial for risk reduction than those who spoke a language other than English. Persons who reported past contact with someone suffering from dementia were more likely to recognise the benefits of mental activity, physical activity and a healthy diet, compared with those who did not have this prior contact.

**Table 6:** Demographic and social factors independently associated with knowledge of risk reduction behaviours

	**Mental activity**		**Social activity**		**Physical activity**		**Healthy diet**		**Moderate alcohol**		**Non-smoking**	
	**Adj OR**	**95% CI**	**Adj OR**	**95% CI**	**Adj OR**	**95% CI**		**Adj OR**	**95% CI**	**Adj OR**	**95% CI**	**Adj OR**	**95% CI**
Sex												
Male (ref)												
Female	1.63	1.22-2.17	1.85	1.24-2.76				1.61	1.19-2.17				
Age group													
20-39 (ref)													
40-59			1.76	1.07-2.89							0.64	0.29-1.42	
60+			3.11	1.85-5.22							0.28	0.09-0.91	
Education													
≤ year 10 (ref)													
Year 11-12/ vocational cert.			0.96	0.53-1.73									
University			2.24	1.29-3.86									
Income													
<$40 k (ref)													
$40-99 k									5.63	1.61-19.75			
$100+									6.69	1.89-23.69			
Language													
Other (ref)													
English	2.52	1.68-3.76						1.79	1.09-2.93	2.48	0.96-6.42		
Dementia contact													
No (ref)													
Yes	1.61	1.17-2.22			1.55	1.13-2.12		1.64	1.14-2.38				

## Discussion

This study has found that many Australians do not recognise dementia as a health priority, and that there is limited understanding of the potential to reduce the risk of this condition through a range of lifestyle behaviours, particularly around mid-life. This indicates that there is great scope to increase awareness that dementia is not an inevitable part of ageing and that, similar to a number of vascular health conditions, the risk of this can be reduced through health promotion action.

Overall, just 7% of respondents rated dementia as one of the top two issues that are important for them to receive information about, which was the same as the proportion of people who nominated “brain health” related conditions as one of their top two issues in the US Brain Health Poll in 2006 [34]. The present study found marked age differences in the perceptions of the importance of dementia, with those aged 60 years and over showing the highest prevalence of concern about this condition. Recent population surveys in other countries, notably France [41] and Israel [42], have reported similar findings that worry and fear of Alzheimer’s disease is significantly higher among older respondents.

Of particular concern is that only 5% of those aged 40–59 years rated dementia as very important, even though they are nearing a stage of life when the risk of the condition increases dramatically and studies show mid-life intervention can be beneficial [43,44]. It may be that this is a reflection of how middle-aged adults rate the severity of, and their personal susceptibility to, dementia relative to other conditions. It was also notable that women were more likely to rate dementia as a priority issue than men. Other researchers have postulated that gender-related patterns such as these may arise from the fact that the prevalence of dementia is higher among women than men [42]. An implication of these findings is that several important population groups may be less likely to attend to information that is disseminated about dementia because of the lower importance they place on this issue [45].

The potential for the risk of dementia to be reduced is central to the public health message about this condition, and while a substantial proportion of Australians were found to have adopted this understanding, the majority are yet to do so. This differs from previous research in Australia [35], which found that 72% of Australians believed the risk of dementia could be reduced, and of the US Brain Health Poll, where 53% responded that there is a lot of potential to improve brain health [34]. An explanation for this is likely to be that in the present study only persons who strongly agreed that the risk of dementia could be reduced were classified as having this understanding, in order to identify those who hold views consistent with the information disseminated by government and non- government agencies.

Reporting prior contact with a person with dementia was independently related to a higher likelihood of understanding that the risk of dementia can be reduced. A plausible explanation of this is that this previous contact has lead to exposure to information about the aetiology and risks factors for this condition, which is consistent with studies which have reported that that health literacy is higher among those with greater contact with health services [46]. Unexpectedly, women were less likely than men to strongly agree that the risk of dementia can be reduced, which is inconsistent with evidence that they have a higher level of health literacy than men [47], and that they more frequently act as primary carers of person with dementia [35]. These gender differences in perceptions about risk reduction may be explained by research concerning the social construction of masculinity and its impact upon health beliefs [48], which has reported that men are socialised to portray independence and control and to deny vulnerability.

The investigation of confidence to reduce the personal risk of dementia was undertaken to determine the extent to which the public not only understand that risk reduction is possible, but believe that it can be successfully applied to their own lives. There was a substantially lower proportion of respondents with this high level of confidence than there was of respondents who understood that risk could be reduced and, once again, women were less likely to convey this view than men. This confidence reflects the outcome expectancies concerning action to reduce dementia risk which is recognised within social cognitive theory as a determinant of goal development and behaviour [49,50]. This finding, therefore, shows a low prevalence of a belief that may be a significant mediator between exposure to public health messages about dementia and personal action.

An encouraging finding was that more than half of respondents believed that efforts to reduce dementia risk should commence before 40 years of age. Respondents over 60 years of age were, however, significantly less likely than those age under 40 years to hold this belief, which may be self-affirmation of their own efforts in later life to maintain brain health. The possible reasons for the lower proportion of respondents from high income households who believed that risk reduction should commence in early adulthood are unclear. This may reflect a view among high income persons that they can draw upon clinical services to address the health problems encountered at different stages of life.

While several studies have investigated knowledge of methods to reduce dementia risk, most have used prompted, closed questions [34,51,52] which may overestimate knowledge prevalence due to the possibility of correct guessing [53]. The unprompted knowledge question used here found that undertaking mentally stimulating activities was the most widely identified activity that a person could do to reduce dementia risk, which is consistent with the findings of several previous studies [35,36,52]. Less than one-third identified physical activity or healthy eating as beneficial, and even lower proportions recognised the role that non-smoking and moderate alcohol intake can play. This shows that there is considerable scope to increase public understanding of the benefits that can be gained for brain health from actions to protect and improve vascular health.

Population segments which showed lower levels of knowledge of several dementia risk reduction behaviours were men, people without prior contact with someone with dementia, and those from a non-English speaking background. There have been few studies which have compared knowledge of dementia risk factors and risk reduction behaviours between ethnic groups. A study in the United Kingdom [54] did not find differences in risk factor knowledge in a convenience sample of older people from south Indian background and Caucasian backgrounds. In a population survey of dementia literacy in Australia, few differences were found in knowledge of dementia risk factors between persons of Greek, Italian and Chinese backgrounds and those who were third generation Australians. Notably, knowledge of mental activity, physical activity and diet did not differ between ethnic groups. In the present study ethnicity was defined according to language preference, and did not distinguish people according to their cultural background or duration of residence in Australia. The lower levels of knowledge of several risk reduction behaviours (mental activity, diet, alcohol moderation) among non-English speaking persons indicates that public education about dementia needs to be undertaken through culturally specific, as well as mainstream media, and in languages other than English.

This study is one of few population surveys of adults across the age-spectrum investigating beliefs and knowledge concerning dementia risk reduction. In order to achieve a representative sample both landline and cell phone numbers were used in random-digit dialling, and almost 60% of those contacted consented to participate. The profile of respondents was similar to that of the Australian population in terms of age, household income and language spoken at home, however, there was an overrepresentation of people who had attained a university level qualification (40.2% vs 25.4% in Australia, [55]. A further limitation was that, while the survey questions had good face validity, their reliability and validity has not been evaluated.

## Conclusions

The growing evidence about the association between dementia and potentially modifiable risk factors, and the significant rise in the global burden of disease due to this condition, has generated wide consensus that a population risk reduction approach must be adopted by policy makers and health promotion agencies. This national survey found, however, that the beliefs and knowledge of adult Australians about dementia are yet to support this perspective. This provides a strong case for dementia risk reduction and brain health to be given greater attention in public health education efforts, and for dementia to be given a higher profile in existing strategies to tackle physical activity, unhealthy eating and other vascular risk behaviors. The data presented here provide a baseline for the measurement of change in public beliefs and knowledge, and identify priority groups for these educational efforts, especially men, people aged less than 60 years, and those from a non-English speaking background.

## Competing interests

The authors declare that they have no competing interests.

## Authors’ contributions

BS managed the study, conducted data analysis and prepared the manuscript. SA assisted in survey design and manuscript preparation. HQ conducted data analysis and reviewed the manuscript. All authors read and approved the final manuscript.

## Pre-publication history

The pre-publication history for this paper can be accessed here:

http://www.biomedcentral.com/1471-2458/14/661/prepub
